# Flavonoids from Engineered Tomatoes Inhibit Gut Barrier Pro-inflammatory Cytokines and Chemokines, *via* SAPK/JNK and p38 MAPK Pathways

**DOI:** 10.3389/fnut.2017.00061

**Published:** 2017-12-18

**Authors:** Matthew L. Tomlinson, Eugenio Butelli, Cathie Martin, Simon R. Carding

**Affiliations:** ^1^Gut Health and Food Safety Research Programme, Quadram Institute, Norwich, United Kingdom; ^2^Martin Laboratory, The John Innes Centre, Norwich, United Kingdom; ^3^Norwich Medical School, University of East Anglia, Norwich, United Kingdom

**Keywords:** diet, flavonoid, inflammatory bowel disease, epithelial, dendritic cell

## Abstract

Flavonoids are a diverse group of plant secondary metabolites, known to reduce inflammatory bowel disease symptoms. How they achieve this is largely unknown. Our study focuses on the gut epithelium as it receives high topological doses of dietary constituents, maintains gut homeostasis, and orchestrates gut immunity. Dysregulation leads to chronic gut inflammation, *via* dendritic cell (DC)-driven immune responses. Tomatoes engineered for enriched sets of flavonoids (anthocyanins or flavonols) provided a unique and complex naturally consumed food matrix to study the effect of diet on chronic inflammation. Primary murine colonic epithelial cell-based inflammation assays consist of chemokine induction, apoptosis and proliferation, and effects on kinase pathways. Primary murine leukocytes and DCs were used to assay effects on transmigration. A murine intestinal cell line was used to assay wound healing. Engineered tomato extracts (enriched in anthocyanins or flavonols) showed strong and specific inhibitory effects on a set of key epithelial pro-inflammatory cytokines and chemokines. Chemotaxis assays showed a resulting reduction in the migration of primary leukocytes and DCs. Activation of epithelial cell SAPK/JNK and p38 MAPK signaling pathways were specifically inhibited. The epithelial wound healing-associated STAT3 pathway was unaffected. Cellular migration, proliferation, and apoptosis assays confirmed that wound healing processes were not affected by flavonoids. We show flavonoids target epithelial pro-inflammatory kinase pathways, inhibiting chemotactic signals resulting in reduced leukocyte and DC chemotaxis. Thus, both anthocyanins and flavonols modulate epithelial cells to become hyporesponsive to bacterial stimulation. Our results identify a viable mechanism to explain the *in vivo* anti-inflammatory effects of flavonoids.

## Introduction

Chronic disease is a growing problem, in terms of direct costs to societies and governments, disability adjusted life years, and quality of life. In 2012, among civilian, non-institutionalized US adults, 49.8% (117 million) had at least 1 of 10 selected chronic conditions (1). Chronic disease is strongly associated with life-style factors, particularly diet (2–5). Fundamental research is required to understand the mechanisms that link diet to health, so that our understanding can go beyond general recommendations, and make predictions, particularly in the form of advice targeted to specific groups of affected individuals.

Natural plant products are known to have anti-inflammatory properties (6), among which are the flavonoids. Flavonoids are specialized polyphenolic compounds abundant in many fruits and vegetables. They have been demonstrated to have several beneficial health effects in age-related diseases, such as cardiovascular disease and cancer (7) and with respect to chronic inflammation; asthma and inflammatory bowel disease (IBD) (8). This study seeks to address the anti-inflammatory mechanisms of action of plant products in a primary cell-based model, using an engineered tomato as a comparative whole food. We hypothesize that flavonoids act primarily on the epithelial layer of the gut to inhibit communication to underlying DCs. This would have the effect of reducing the level of acute gut inflammation becoming chronic.

Inflammatory bowel disease describes two idiopathic chronic inflammatory conditions of the gut: Chron’s disease and ulcerative colitis. The etiology of IBD remains unknown, although both conditions are usually considered multifactorial disorders arising from a breakdown in mucosal immune tolerance to resident intestinal microbes (9). IBD is characterized by alternating phases of clinical remission and relapse (10). It is a growing global health burden and is often associated with industrialization, with 0.5–1% of the UK population being affected (11). A variety of pharmacological and surgical treatments exist with prescription based on disease severity (12). However, these treatments are often life-long and may have a limited efficacy and detrimental side effects (13). Dietary intervention as an IBD therapeutic is an attractive approach as it could be used to not only attenuate the immune system, potentially making it hyporesponsive, but also because high topological doses and absence of side effects of normal dietary components could potentially promote longer remission phases. Promotion and maintenance of patient remission remains a long-term goal in IBD therapeutics, which is where preventative actions, especially dietary changes could have a real impact.

The effects of flavonoids on chronic gut inflammation have been demonstrated in animal-based models (14) and recently in clinical trials (15). Positive effects include, reduction of bleeding, improvement in stool consistency, improved colon histological appearance, decreased weight loss, and protection from colon shortening in rodent studies (14). Flavonoids are likely to be pleiotropic (16). However, several potential molecular targets have been proposed, including key inflammatory signaling pathways (17). This has an advantage over drugs designed to single targets, potentially countering the immune system’s high degree of redundancy.

Many immune cells play a role in IBD, our study focused on the boundary epithelial cells and their interaction with mucosal dendritic cells (DCs) and in particular on the cytokines and chemokines that mediate interactions between the epithelium and DCs. DCs provide the link between the innate and adaptive immune systems. Intestinal inflammation is characterized by the influx and accumulation of activated immune cells, in particular DCs, into the gut mucosa (18) with increased numbers of DCs believed to be central in the pathogenesis of colitis (19).

In parallel to reducing chronic inflammation, healing of the gut mucosa is regarded as an important factor in the treatment of IBD (20). This healing is driven by the STAT3 kinase signaling pathway (21). Mucosal healing occurs in several stages, firstly by epithelial restitution where adjacent epithelial cells migrate to cover the wound area (22). Then epithelial cell proliferation increases the cellular pool at the wound site, followed by differentiation (22). Any proposed therapeutic treatment for IBD needs to demonstrate no adverse effects on the processes of healing or cause aberrant apoptosis, to be effective in long-term disease management.

Plant biotechnology offers methods for targeted enhancement of the levels of different phytonutrients in widely consumed plant foods, such as tomatoes. Many health-benefit studies have been confounded because purified phytochemicals fail to have the same effects as they do in food. These problems are avoided by addressing the effects of each phytonutrient and combinations at equivalent levels in a matched food matrix. Our study takes a unique approach, by using an engineered food as a comparative tool to study the anti-inflammatory effects of diet.

## Materials and Methods

### Preparation and Analysis of Aqueous Tomato Extracts

Aqueous tomato extracts were prepared from *Del/Ros1N* (H-antho) and *AtMYB12* (H-flav) lines (23). After homogenization, supernatants were clarified by centrifugation, aliquoted (single use), and stored at −80°C. To quantify the major phenolics present in the tomato extracts, we used the following phenolics as standards: chlorogenic acid, rutin, kaempferol rutinoside, quercetin, and cyanidin. Samples were analyzed on a Prominence/Nexera UHPLC equipped with an ion-trap ToF mass spectrometer (Shimadzu). Phenolics were separated on a 100 × 2.1 mm 2.6 μ Kinetex EVO C18 column (Phenomenex) using the following gradient of acetonitrile (solvent A) versus 1% formic acid in water (solvent B) run at 500 µL min^−1^ and 40°C: 0 min, 2% A; 0.5 min, 2% A; 5 min, 10% A; 17 min, 30% A; 25 min, 90% A; 25.8 min, 90% A; 26 min, 2% A; 30.1 min, and 2% A. Detection was performed using UV/visible absorbance by collecting spectra from 200 to 600 nm at 6.25 Hz, and by positive mode electrospray MS. Flavonoids were quantified from UV absorbance at 350 nm, chlorogenic acid at 325 nm, and both with bandwidth of 4 nm. Anthocyanins were quantified from visible absorbance at 500–550 nm, following subtraction of a reference of 590–600 nm. MS data were used to confirm the presence and identity of compounds quantified by UV/visible absorbance.

### Cell Isolation and Culture

Male C57BL/6 mice at 6–8 weeks of age were used throughout. Murine colonic epithelial cell (CEC) isolation and culture were performed as described in Baumgart et al. (24). Briefly, colons were isolated, cleaned, and the epithelial layer dissociated by selective protease digestion. CECs were cultured for 3 days at 37°C with 5% CO_2_ and 95% air. CECs were plated at a density of 1.5 × 10^6^ CEC cells per well in a 6-well plate with 2 mL of media, with or without the addition of aqueous tomato extracts (each repeat is a pooled sample of five biological replicates). Cell preparations were assayed for epithelial cell enrichment by staining with cytokeratin (Sigma F3418) and analyzed by flow cytometry (Beckman Coulter FC500).

### Cytokine and Chemokine Analysis

Cells were incubated with aqueous tomato extracts, added to a 2% final concentration (volume/volume ratio), 40 µL in 1,960 µL of media. After 4 h of incubation with tomato extracts, they were microbe-associated molecular pattern (MAMP) stimulated—1 µg/mL of peptidoglycan (*Staphylococcus aureus*, Sigma), 1 µg/mL of muramyl dipeptide (Bachem), and 1 µg/mL of lipopolysaccharide (*Salmonella typhosa*, Sigma). After 3 days of culture, the conditioned media were clarified by centrifugation.

Murine trans-immortalized intestinal cell-line (m-IC_cl2_) cells were used as a model of a polarized gut barrier, grown to a confluent monolayer and incubated for 4 h prior to stimulation, with and without tomato extracts. Aqueous tomato extracts were added to a 2% final concentration (v/v). The conditioned media were collected 24 h post-stimulation.

Conditioned media from both cell types were then analyzed using cytometric bead arrays (CBA, BD Bioscience), according the manufacturer’s instructions. Three replicates of each experiment were assayed. Mean fluorescence intensity values were converted to pg/mL by polynomial regression analysis, calculated from standard curves generated from recombinant protein standards. The mean values and SD were calculated from three replicates. Each replicate is a pooled sample of five mice. Key inflammatory chemokines were selected based on their expression in mouse and human CECs (25, 26) and their upregulation in experimental mouse colitis models and human IBD patients (23, 27–29).

### Chemotaxis Assays

A transwell (Boyden chamber) assay was used to determine the effect of reduced chemokines on the movement of primary murine DCs, with recombinant chemokines at the same concentrations, to replicate control and flavonoid addition. The following recombinant chemokines were added to the lower chambers to mimic the results seen with CECs and 2% aqueous wild-type extract: CCL2—3,827 pg/mL (R&D Systems 479-JE); CXCL1—2,406 pg/mL (R&D Systems 453-KC); CCL5—228 pg/mL (R&D Systems 478-MR); CCL3—136 pg/mL (R&D Systems 450-MA-010); and CCL4—125 pg/mL (R&D Systems 451-MB). To mimic the results seen with CECs and the average value with 2% high-flavonol tomato extract addition (CCL2—789 pg/mL), chemokines were diluted in serum free Hanks’ Balanced Salt solution. Mouse spleen polymorphonuclear leukocytes (CD45^+^, BD Bioscience 563890 and isotype control BD Bioscience 562603) were isolated according to Inaba et al. (30) with a optiprep-based flotation protocol (Axis-shield) and added to the top chamber of 5-µm pore size inserts (Costar) in 24-well plates. Fluorescence-activated cell sorting (FACS) gave a 30–40% enrichment of DCs. Using live staining with both CD11c^+^ (BD Bioscience 560583 and isotype control BD Bioscience 560555) and MHC-II^+^ antibodies (BD Bioscience 562366 and isotype control BD Bioscience 560784). For each assay the cells were pooled so that DC numbers were consistent.

Chemotaxis assays were incubated at 37°C for 2 h. Chemotaxis assays were replicated three times. Each replicate was a pooled sample of mouse spleen polymorphonuclear leukocytes from 10 mice. Cell numbers in the lower chambers were counted by FACS as well as by hemocytometer. For hemocytometer counts, total numbers of cells were counted three times for each sample and mean values and SD around the mean were calculated. For FACS analysis, the entire content of the lower chamber was stained to detect DCs with the following antibodies: CD45, CD11c, and MHCII. Half the lower chamber volume was assayed by FACS and DC cell counts were extrapolated back to the original volume. Some of the MHCII^+^/CD11C^+^ labeled cells might have included a macrophage subset.

To allow a checkerboard analysis matrix to be generated, recombinant chemokines were added to either upper or lower chambers, or both at previously stated concentrations, to discern between chemokinesis and chemotaxis. Serum free HBSS was added to the chambers as a media-only control. The following gating strategy for FACS analysis was used; single-cell events were gated for using forward and side scatter parameters. Following this, unstained cells, isotype (to determine non-specific antibody binding) and fluorescence minus one controls (to determine gating boundaries in multicolor flow cytometry experiments) were run. A bivariate histogram was used to determine the gating for each cell type.

### Phosphorylation State of Inflammation Cascade Kinases

An ELISA assay (Cell Signaling Technology 7276) containing members of key inflammatory signaling pathways, including NF-κB, SAPK/JNK, p38 MAPK, and STAT3, was used. Primary murine CECs were isolated as previously described (24) and normalized to 1.5 × 10^6^/mL by hemocytometer and cultured for 4 h in the presence or absence of tomato extracts. Aqueous tomato extracts were added to a 2% final concentration (volume/volume ratio). The cultures were then stimulated with bacterial peptides (MAMPs); 1 µg/mL of peptidoglycan (*S. aureus*, Sigma), 1 µg/mL of muramyl dipeptide (Bachem), and 1 µg/mL of lipopolysaccharide (*S. typhosa*, Sigma) for 30 min, except the uninduced control. The assay was replicated three times. Stimulated cells were then lysed immediately following the protocol recommended by the manufacture. Protein concentrations were checked by Lowry protein assay (Bio Rad 500-0120), and samples were normalized to 700 µg/mL. Cell lysates were diluted 1:1 with sample dilutant (Cell Signaling Technology) and 200 µL of diluted lysates were used per well.

To determine if the ELISA results were significantly different, single-factor ANOVA analysis was performed.

### Wound Healing (Gap Closure Assays)

The m-IC_cl2_ cell line was cultured in 12-well plates and serum starved for 24 h, pre-assay. Cells were incubated with aqueous tomato extracts at 2% final concentration (v/v) for 4 h then a single scratch was made to the confluent monolayer with a P1000 pipette tip. Wounded monolayers were washed three times with serum-free media. Assays were replicated three times. Microscopy images were taken on a Zeiss Axiovert 200 M at time 0 h, and scaled wound width measurements were taken with Zeiss Axiovision 4 software. A permanent mark on the underside of each well, intersecting a scratched area, acted as a reference point. Cells were incubated for a further 18 h with and without tomato extracts and measurements taken again at the reference point.

To investigate the effects of flavonoids on wound-associated proliferation, BrdU incorporation assays were performed. BrdU labeling reagent (Zymed 00-0103) was added at a 1:100 concentration at T-0 and then removed with a media change (X3) 3 h later. These assays were also replicated three times. Cells were fixed at 18 h and immunohistochemistry (anti-BrdU antibody, BD Bioscience 347583) followed a paraformaldehyde/saponin-based staining procedure as described in Rothaeusler and Baumgarth (31). Images taken from five replicates were visually scored for BrdU-stained cells.

### Cell Viability, Proliferation, and Apoptosis

To investigate the effects of flavonoids on cell viability, primary murine CECs were cultured with and without tomato extracts at 2% final concentration (v/v) for 3 days. In addition, CECs were cultured for 18 h to mimic the conditions in the wound healing assays. m-IC_cl2_ cells were trypsin treated to dissociate the cells and gently pipetted to maintain membrane integrity and minimize false-positive results. For CEC and m-IC_cl2_ assays, two antibody markers of early apoptosis were used, annexin V (BD Bioscience 556570) and active Caspase 3 (BD Bioscience 550914) and a propidium iodide exclusion assay (BD Bioscience 556570) for non-viable cells. Actively proliferating cells were stained with Ki-67 (BD Bioscience 562899 and isotype control BV480), repeated three times. BrdU incorporation assays were performed as previously described. For both cell types, the assays were repeated three times. Stained cells were assayed by flow cytometry, according to the manufacturer’s protocols. The following gating strategy for flow cytometry analysis was used: single-cell events were gated for using forward and side scatter parameters. Following this, unstained cells and isotype controls were run. All flow cytometry and previously mentioned FACS data were analyzed using FloJo 7 software (Tree Star, Inc.). Mean values and SD were calculated from three replicates.

### Statistical Analyses

Excel was used as the statistical software for the data analyses. For comparing two groups, Student’s *t*-test were used. These assumed unequal variances to determine which of the mean values were significantly different with a hypothesized mean of 0 and an alpha value set at 0.05 (the null hypothesis was rejected if the two-tailed *p*-value was <0.05). For comparing multiple groups, one-way ANOVA tests were used. using a 0.05 alpha setting. A null hypothesis was rejected if the *F*-value > *F* critical value. Statistically significant results (*p* < 0.05) are indicated with an asterisk symbol (*) in the figures. Non-significant results (*p* > 0.05) are indicated with a hash symbol (^#^).

### Ethical Considerations

Animal experimentation was performed with University of East Anglia Animal Procedures Ethics Committee and UK Home Office approval (Protocol no. PPL 40/6325, 80/2355).

## Results

### Analysis of Engineered Tomato Flavonoid Content

The engineered tomatoes have been described before (23, 32) and the concentrations of the principal components in the wild-type, high-anthocyanin, and high-flavonol extracts used in this study are given in Table 1. The high-anthocyanin extract showed a significant enrichment in the anthocyanin delphinidin and small increases in the flavonol rutin and the other components tested, including chlorogenic acid (Table 1). The high-flavonol extract showed substantial increases in glycosylated flavonols and chlorogenic acid levels (Table 1) as compared with the control (wild type) tomatoes. Aside from differences in concentrations of different flavonoids, the high flavonol extract had a higher overall flavonoid content (4943.26 µM) compared to the high-anthocyanin extracts (641.81 µM), see Table 1.

**Table 1:** Quantification by LC/MS analysis of the most abundant polyphenols in aqueous extracts of high-anthocyanin (H-antho), high-flavonol (H-flav), and control wild-type (WT) tomato fruit.

Compound	Wild Type	H-antho	H-flav
CGA	72.35 (15.96±)	197.06 (3.16±)	472.63 (9.59±)
KaeRut	10.86 (0.16±)	47.27 (0.72±)	2853.56 (52.63±)
Rut	62.62 (4.39±)	99.86 (3.63±)	1021.31 (16.58±)
KaeRutGlc	0	4.25 (0.61±)	443.67 (8.08±)
KaeGlc	6.22 (0.46±)	12.54 (0.56±)	561.66 (16.62±)
QueRutGlc	5.43 (0.51±)	1.36 (2.36±)	63.09 (0.27±)
DelCouRutGlc PetCafRutGlc*	0	217.96 (1.32±)	0
PetCouRutGlc	0	238.46 (1.61±)	0
DelCafRutGlc	0	20.11 (0.40±)	0

*All values expressed as micromolar. Mean values are shown with ± SD. CGA, chlorogenic acid; KaeRut, Kaempferol-rutinoside; Rut, Rutin; KaeRutGlc, Kaempferol-rutinoside-glucoside; KaeGlc, Kaempferol-glucoside; QueRutGlc, Quercetin-rutinoside-glucoside; DelCouRutGlc, Delphinidin-glucoside-coumaroyl-rutinoside; PetCafRutGlc, Petunidin-glucoside-caffeoyl-rutinoside; PetCouRutGlc, Petunidin-glucoside-coumaroyl-rutinoside; DelCafRutGlc, Delphinidin-glucoside-caffeoyl-rutinoside*.

*The DelCouRutGlc MS peak also contains traces of a minor anthocyanin, PetCafRutGlc

### Flavonoids Specifically Inhibit Gut Barrier Pro-inflammatory Cytokine Secretion

**Figure 1:**
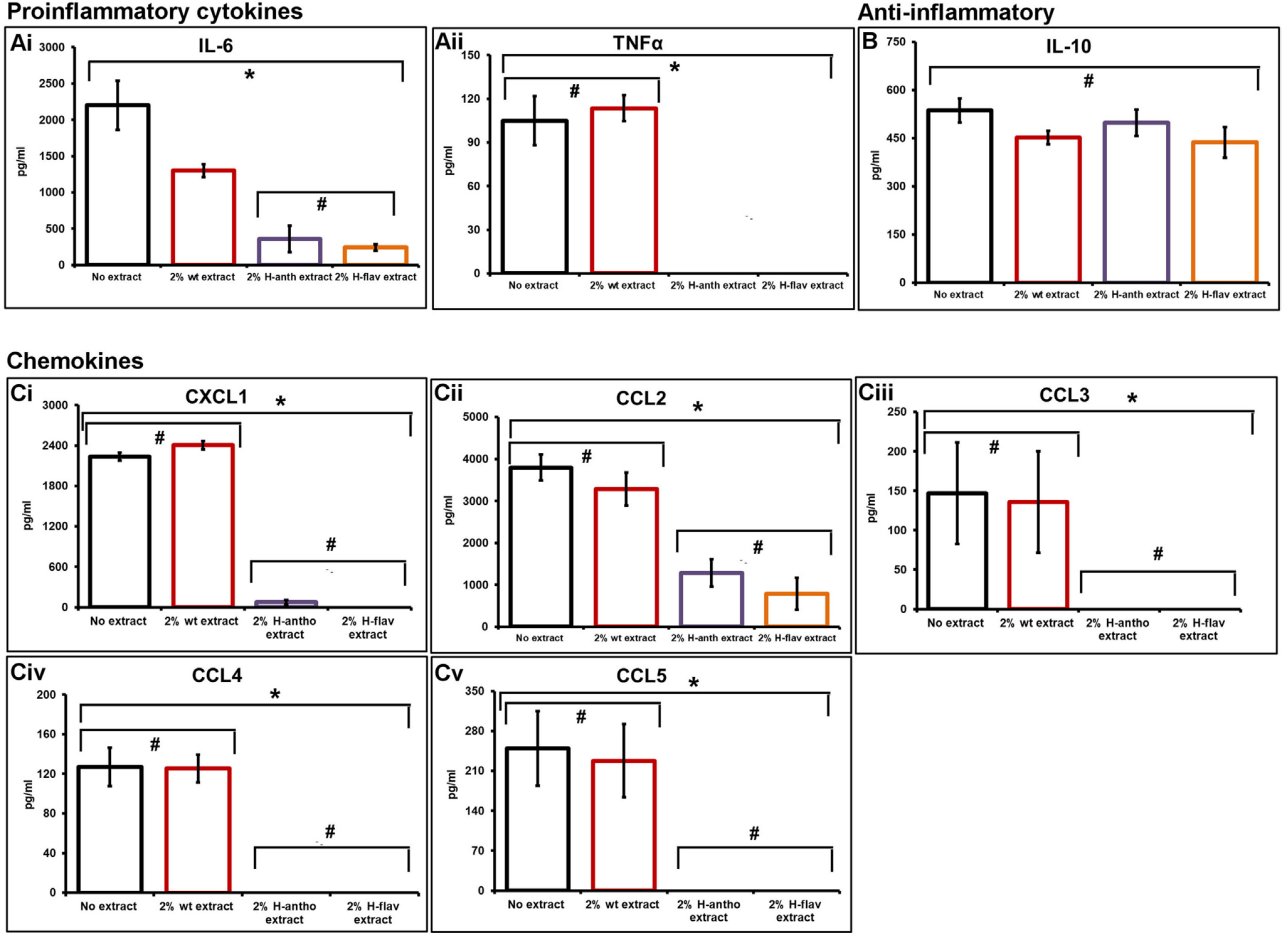
Flavonoid-enriched tomato extracts inhibiting key pro-inflammatory cytokines and chemokines. An inflammatory response in primary murine colonic epithelial cells was initiated with microbe-associated molecular patterns, with and without tomato extracts. Cytometric bead arrays and flow cytometry was used to assay secreted cytokine and chemokine levels. Pro-inflammatory cytokines [**(A)**, i,ii], anti-inflammatory cytokine IL-10 **(B)**. A set of inflammatory bowel disease chemokines were inhibited [**(C)**, i–v]. Y-error bars show SD values around the mean, *n* = 3. *Statistically significant (*p* < 0.05), ^#^Not statistically significant (*p* > 0.05).

As the colonic epithelium receives the highest dose of dietary flavonoids, we investigated their effect on epithelial barrier cytokine secretion using a primary murine CEC inflammation model. CECs were cocultured with several MAMPs: lipopolysaccharide, peptidoglycan, and muramyl dipeptide to initiate a broad inflammatory response in the presence or absence of tomato extracts. Using cytometric bead arrays, we assayed several key inflammatory cytokines from the same sample. The culture of CECs without MAMP stimulation produces a very low level of cytokine production (<10 pg/mL). Analysis showed that the addition of wild-type tomato extracts reduced IL-6 levels by 41% compared with the no extract controls (Figure 1Ai). However, the addition of the high-anthocyanin and the high-flavonol extracts had a pronounced inhibitory effect, with 84 and 90% IL-6 reduction, respectively (Figure 1Ai). TNFα secretion was also reduced to undetectable levels by addition of either high-anthocyanin or high-flavonol tomato extracts (Figure 1Aii). The levels of IL-10 with both high-anthocyanin and high-flavonol tomato extract addition remained comparable with values for control and wild-type tomato extract values (Figure 1B). Statistical analysis (ANOVA) showed that there were no significant effects of addition of different tomato extracts (*p* < 0.05) on IL-10 secretion (Figure 1B). ANOVA analysis of the pro-inflammatory cytokines showed a significant difference (*p* < 0.05) compared with no extract and wild-type extract addition.

### Flavonoids Inhibit the Epithelial Secretion of a Set of IBD Chemokines

Addition of wild-type tomatoes had no effect on chemokine secretion (Figure 1Ci–v). The addition of the high-anthocyanin tomato extract inhibited the secretion of CXCL1 by 97%, and also completely inhibited CCL3, CCL4, and CCL5 secretion (Figure 1C). Complete inhibition of CXCL1, CCL3, CCL4, and CCL5 was also observed with the addition of the high-flavonol tomato extract (Figure 1C). Both the high-flavonol and high-anthocyanin tomato extracts reduced CCL2 levels by 67 and 80% for anthocyanins and flavonols, respectively (Figure 1Cii).

### Lower Epithelial Chemokines Lead to Reduced Leukocyte and DC Chemotaxis

**Figure 2:**
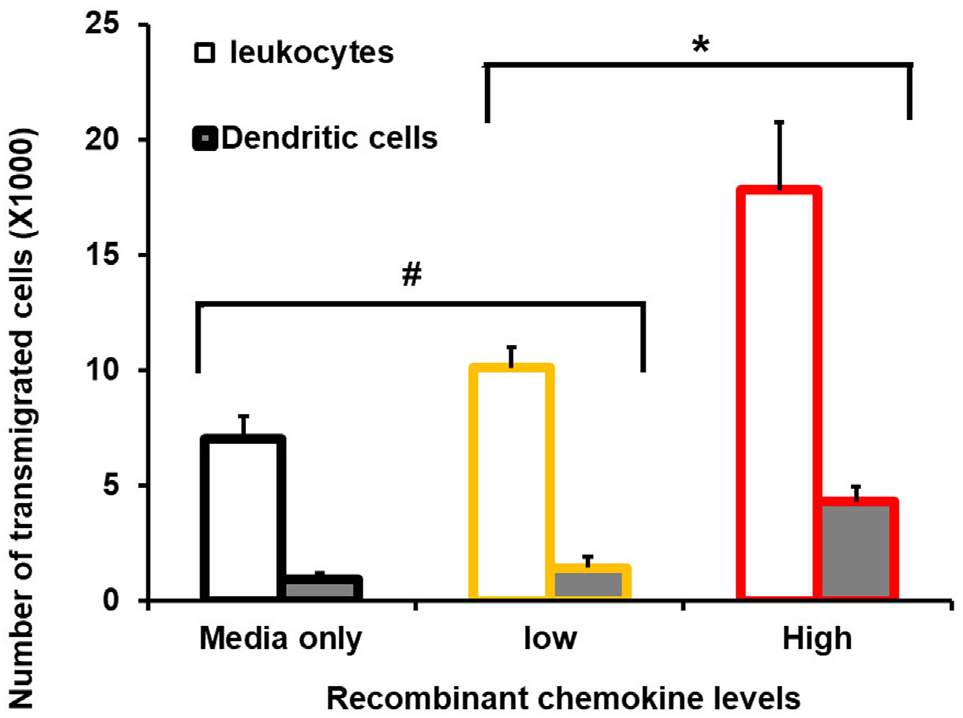
Consequence of reduced chemokine levels on primary murine leukocyte and dendritic cell (DC) migration. FACS-sorted primary cells and Boyden chambers assays were used. Average number of transmigrated primary murine spleen CD45^+^ (leukocytes) and primary spleen CD11C^+^/MHCII^+^ (DCs) are shown. The *X*-axis shows zero, no recombinant chemokines in the lower Boyden chamber (media-only control), and low levels replicated the mean value with 2% high-flavonol tomato extract addition. High represents colonic epithelial cells, induced with microbe-associated molecular patterns, in the presence of wild-type tomato extract. Y-error bars show SD values around the mean, *n* = 3. *Statistically significant (*p* < 0.05). ^#^Not statistically significant (*p* > 0.05).

Transwell migration (Boyden chamber) assays revealed an increased number of leukocytes (61%) migrating toward a high-chemokine gradient. High chemokines (Figure 2) represent CECs, induced with MAMPs, in the presence of wild-type tomato extract. Importantly, a proportionally larger number of DCs had transmigrated (78%) than in the media-only control (Figure 2). However, this migration was reduced with low-chemokine levels, representing engineered tomato extract addition (based on average chemokine levels with 2% high-flavonol tomato extract addition) (< 30% leukocytes and < 33% DCs as compared with the media-only control). The differences between the low- and high-chemokine groups were found to be significant (*p* < 0.05, *t*-test). Checker board analysis revealed a strong chemotactic and not a chemokinetic response (Table 2).

**Table 2:** Checker board analysis of Boyden chamber assays.

	Upper chamber		
**Lower chamber**		0	High
	0	1 (9.85%±)	0.61 (10.79%±)
	High	2.56 (15.24%±)	1 (8.59%±)

*Ratios of counted cells, both by hemocytometer and FACS. 0 values refer to serum free HBSS only chamber, High refers to the levels of chemokines seen with MAMP stimulated colonic epithelial cells and 2% wild-type aqueous extract. n = 3. Mean values are shown with SD*.

### Flavonoids Suppress Several Pro-inflammatory Epithelial Signaling Pathways

To investigate potential flavonoid molecular targets, we assayed key inflammatory kinase signaling pathways known to play a role in IBD in both human and rodent studies (33–35). An ELISA assay with phospho-epitope specific capture antibodies was used, along with primary murine CEC lysates, to assay the activation state of NF-κB, SAPK/JNK, p38 MAPK, and STAT3 kinases.

**Figure 3:**
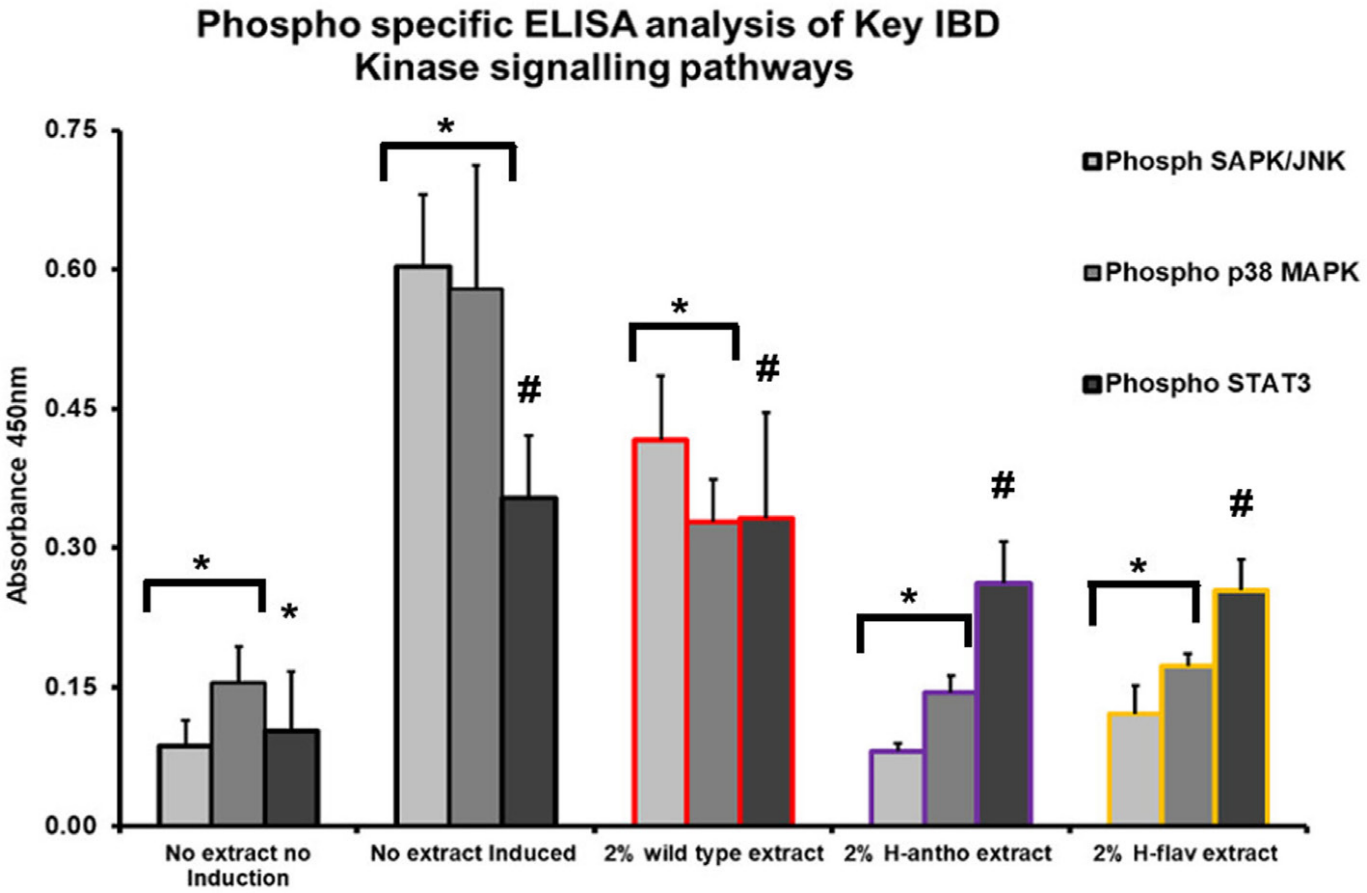
Phospho-specific ELISA assay used with primary murine colonic epithelial cells, induced inflammatory response *via* microbe associated molecular pattern (MAMP) addition. Tomato extracts were added prior to MAMP addition. Y-error bars show SD values around the mean, *n* = 3. *Statistically significant (*p* < 0.05). ^#^Not statistically significant (*p* > 0.05).

Colonic epithelial cells treated with wild-type tomato extract showed a small, but significant decrease in SAPK/JNK activity (Figure 3). High-flavonol extracts dramatically reduced the activity of SAPK/JNK (Figure 3), and high-anthocyanin tomato extract reduced the activity of SAPK/JNK by 87% (Figure 3) as compared with no tomato extract controls. These differences were all found to be significant (*p* < 0.05, ANOVA analysis and *t*-test). The high-flavonol extract reduced p38 MAPK activation by 70% as compared with the no-tomato extract control (Figure 3). High-anthocyanin extracts reduced p38 MAPK activation by 75% (Figure 3). Wild-type extract inhibited p38 MAPK activation by 43% (Figure 3). The wild-type extract had a significant effect on p38 MAPK activation as did high-anthocyanin and high-flavonol extracts. The high flavonol significantly inhibited p38 MAPK phosphorylation more than the high anthocyanin extract. These differences were found to be significant (*p* < 0.05, ANOVA and *t*-test).

None of the tomato extracts showed a significant inhibition of the intestinal wound healing-associated STAT3 pathway (*p* < 0.05, ANOVA) (Figure 3). However, STAT3 activation was significantly induced with MAMP induction (Figure 3).

### Flavonoids Do Not Effect Intestinal Wound Healing

**Figure 4:**
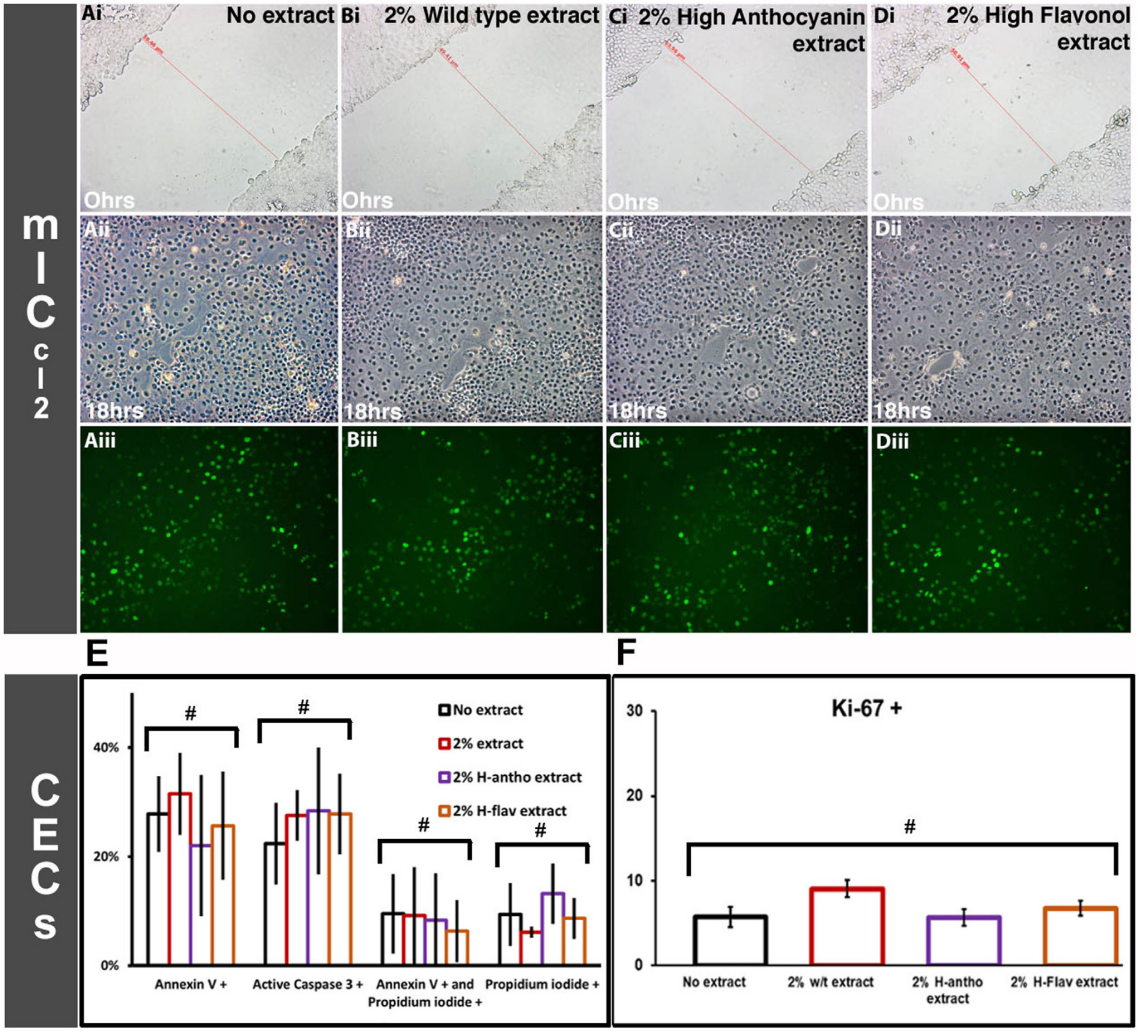
Effect of flavonoid-enriched tomato extracts on intestinal wound healing. [**(A–D)**, i] cellular migration m-IC_cl2_-based cell assays at time Ohr scratch wounds. [**(A–D)**, ii] The same scratched areas at 18 h post wounding. [**(A–D)**, iii] The same scratched areas as [**(A–D)**, ii], but with BrdU staining (proliferating cells). Representative images are shown. Colonic epithelial cell based assays for apoptosis and proliferation. **(E)** Apoptotic and non-viable CECs after 3 days of flavonoid-enriched tomato extract addition at 2%, *n* = 3. **(F)** Proliferating (Ki-67+) CECs after 3 days coculturing with tomato extracts at 2%, *n* = 3. Y-error bars show SD values around the mean, *n* = 3. ^#^Not statistically significant (*p* < 0.05).

As none of the high-flavonoid tomato extracts affected activation of the STAT3 pathway (Figure 3), we investigated the processes linked to STAT3 function further, by looking at apoptosis, cellular proliferation, and migration. Scratch wound assays (m-IC_cl2_ cells) were used to investigate cellular migration at a wound site. CECs are semi-adherent, as such scratch wound assays could not be performed. Addition of 2% wild-type tomato extract or either high-anthocyanin or flavonol tomato extract addition had no effects on m-IC_cl2_ cell migration (Figures 4Ai,ii–Di,ii; Figure S1 in Supplementary Material). BrdU incorporation assays on the m-IC_cl2_ monolayer showed that addition of either flavonoid-enriched extract had no effect on cellular proliferation at 2% (Figures 4Aiii–Diii). Figure 4E shows that at 2% the tomato extracts had no effect on either CEC apoptosis or cell viability (*p* < 0.05, ANOVA). Figure 4F shows that they also had no adverse effect on CEC proliferation (*p* < 0.05, ANOVA). Tomato extracts enriched with flavonoids at 2% had no significant effect on apoptosis or cell viability after 3 days of m-IC_cl2_ culture (Figure S2 in Supplementary Material) (*p* < 0.05, ANOVA). In conclusion, flavonoids had no observed effects on the wound healing processes associated with IBD remission.

## Discussion

We have taken a unique approach to assay the anti-inflammatory actions of flavonoids, using tomatoes engineered to express high levels of flavonoids (either anthocyanins or flavonols) to allow comparative analysis with an isogenic whole food matrix (32). Previous studies have focused on single polyphenol standards or whole foods with no-comparable control (36). The aqueous tomato extracts do not include the hydrophobic carotenoid lycopene, allowing the flavonoid effects to be assayed independently of this known biologically active compound, normally in tomatoes.

Our study used transient cultures of primary mouse CECs that have been demonstrated to release a wide range of cytokines and chemokines in response to bacterial antigen stimulation (26), in an analogous way to human CECs (37). They also receive the highest topological doses of dietary flavonoids in the body (14). Flavonoids are known to be readily absorbed from the gut lumen and taken up at the apical surface of the epithelial monolayer *via* active transport (38). Interestingly, a previous study has concluded that there was no further translocation across the basolateral side, to the lamina propria (39), indicating flavonoid accumulation in epithelial cells.

For the results presented in our study to have a clinical impact, the flavonoids must be at achievable levels. Estimates of flavonoid levels consumed in the human diet range from 20 to 1,000 mg/day (40) with the mean intake in Europe estimated to be 428 mg/day (41). Ingestion of 500 mg of polyphenols has been estimated to give local intestinal concentrations of about 300 µM (42). Rutin administered to rats at 10 mg/kg accumulated to a local concentration in the large intestine at 220 µM (43). We believe that the levels of flavonoids used in this study are achievable by dietary means, although more extensive pharmokinetic analysis of different dietary flavonoids would be needed to support this interpretation.

The MAMPs in our CEC inflammation model have been previously shown to initiate CEC secretion of IL-6 and CCL2 (26). The effect of flavonoids on chemokine secretion has been demonstrated for individual chemokines, such as IL-8 (the human equivalent to mouse CXCL1) and CCL2 (44), TNFα and IL-6 (15). Of particular interest is the ability of both the high-flavonol and the high-anthocyanin tomato extracts to inhibit TNFα. Anti-TNFα immunotherapy is an effective treatment for IBD (45). TNFα has been shown in macrophages to be inhibited by flavonoids (46). Quercetin has been shown to decrease CCL3 release from adipocytes and macrophages (47) and flavonoid inhibition of CCL4 and CCL5 secretion was observed in a study with macrophages infected with *Chlamydia trachomatis* (48).

The value of using engineered tomatoes as a complex comparative food model is demonstrated by the inhibition of IL-6 by the control wild-type tomato extract (Figure 1Ai). This would have been missed using polyphenol standards or whole foods without a comparative control. Missing the effects of the food matrix could lead to a possible overestimation of cytokine inhibition. The high-flavonol tomato extracts contained significantly higher amounts (over sevenfold) of total flavonoids compared with the high-anthocyanin extracts. This may explain the greater efficacy of the high-flavonol extracts at reducing markers of CEC inflammation. However, anthocyanins may actually have a higher efficacy, because similar effects were seen with significantly lower concentrations.

Analysis of the CEC produced Th2 immunoregulatory chemokine IL-10, revealed no effect with flavonoid exposure (Figure 1B), consistent with other studies (15), and suggests specific effects of flavonoids in repressing pro-inflammatory cytokines. This is of importance since IL-10 plays a key role in IBD, by inhibiting pro-inflammatory responses (49).

Assaying the activation state of several key pro-inflammatory signaling pathways revealed a significant inhibition of p38 MAPK phosphorylation by all the tomato extracts, including extracts of the wild-type comparator (Figure 3). However, significantly stronger effects were observed with both the high-anthocyanin and high-flavonol tomato extracts (Figure 3). p38 MAPK is believed to play a central role in the regulation of a wide range of immunological responses, including pro-inflammatory responses (50) and in IBD (51). In intestinal epithelial cell lines, p38 activation regulates IL-8 expression (the mouse homolog of which is CXCL1) (52). CXCL1, CCL2, and CCL5 secretion is also linked to p38 MAPK activation in intestinal epithelial cells (53, 54). The SAPK/JNK kinase pathway also plays a key role in regulating pro-inflammatory responses in IBD (55). SAPK/JNK is known to regulate CCL5 expression in intestinal epithelial cells (56) and IL-6 expression in the colon (57). Of the major intracellular signaling cascades examined (NF-κB, p38 MAPK, ERK, and SAPK/JNK), we found inhibition of the activation state of two; p38 MAPK and SAPK/JNK. The ERK pathway was not investigated because its primary role in inflammation is in T-cell activation (58).

Considering that anthocyanins and flavonols cause comparable biological effects, it is likely that they target similar steps in key pro-inflammatory signaling pathways. It remains unclear whether flavonoids exert their biological effects by interactions with cell surface proteins or cytoplasmic/nuclear proteins, and this is an area requiring further investigation. As flavonoids are thought to be pleiotropic, their effects are could involve a combination of molecular targets.

A commonly cited flavonoid molecular target is the NF-κB kinase pathway (17). We did not observe significant levels of the NF-κB p65 subunit or its inhibitor IκBα, or phosphorylated levels of NF-κB p65 in our CEC ELISA-based assays. This is consistent with evidence from studies using NF-κB^EGFP^ transgenic mice which shows NF-κB expression in the colon to be confined to lamina propria mononuclear cells (59). NF-κB in CECs has been reported to be involved in intestinal homeostasis, including maintaining epithelial integrity, and not in any pro-inflammatory response (60). TLR signaling (as in an MAMP-induced inflammatory response) in CECs also does not involve NF-κB activation (61).

We did not observe an inhibition of the STAT3 kinase pathway. STAT3 activation is involved in intestinal barrier maintenance, *via* regulation of epithelial cell proliferation and survival (62). This provides a molecular explanation as to why no effect on apoptosis, proliferation, or migration was seen with flavonoid addition (see Figure 4). STAT3 is also important for the efficient regeneration of the epithelium in response to injury (63). In mice, STAT3 activity co-localizes to proliferating intestinal crypt cells near the wound site (21).

Our study highlights the inhibitory effects of flavonoids on cytokine and chemokine release and the inhibition of the major inflammatory kinase pathways induced in inflammation and the consequences for DC migration. Under homeostatic conditions, a steady-state population of DCs patrols the epithelium, acting as sentinel cells, to promote tolerance (64). However, recruitment of large numbers of activated DCs in IBD from the lamina propria to the epithelium is a chemokine-driven process (64). First, enterocytes (the main epithelial cell) along with sentinel DCs sense bacterial-derived antigens (MAMPs) *via* pattern recognition receptors such as toll-like receptors (TLRs) and NOD2, then secrete chemokines to rapidly recruit more DCs (64). Epithelial cells also recruit unconditioned monocytes that develop into inflammatory DCs for the induction of immunity (65). DCs have a vital role in IBD, demonstrated when DCs are ablated during DSS-induced colitis and disease manifestation is ameliorated (9). Further evidence comes from colitis being associated with an increase in DCs in the colonic mesenteric lymph nodes and blocking interactions between DCs and T cells prevents colitis (9).

A proposed model of DC migration following exposure to high-flavonoid levels is shown in Figure S3 in Supplementary Material. To the best of our knowledge, this is the first time that the inhibition of a set of key IBD chemokines has been demonstrated by flavonoids from the epithelial barrier (using primary cells, instead of immortalized cell lines). Our results highlight a mechanism by which flavonoids could exert their effects in making the epithelial barrier hyporesponsive, by reducing the responsiveness of key pro-inflammatory kinase signaling pathways to the microbiota in a genetically susceptible individual. This should help direct future *in vivo* and human intervention studies. We show the benefits of using engineered tomatoes as a research tool in contrast to purified standards, or whole foods rich in a diverse number of biologically active compounds that have no comparator control.

The role of diet in modulating the state of the immune system is an area with huge potential to improve the quality of life of those living with chronic conditions and to reduce some of the burden imposed on our health system.

## Ethics Statement

Animal experimentation was performed with University of East Anglia Animal Procedures Ethics Committee and UK Home Office approval (Protocol no. PPL 40/6325, 80/2355).

## Author Contributions

MT, SC, and CM were involved in the conception and design of the study. MT performed data acquisition, analysis, and interpretation. EB organised HPLC/Mass spec analysis. MT wrote the manuscript. MT, SC, and CM contributed to revisions and intellectual input.

## Conflict of Interest Statement

The authors declare that the research was conducted in the absence of any commercial or financial relationships that could be construed as a potential conflict of interest.

## Supplementary Material

Figure S1 Effect of flavonoids on the extent of wound closure on an m-ICcl2 cell line-based scratch wound model, percentage closure calculated by wound diameter at T-0 compared with the same wound area at T-18 h using scaled wound width measurements. Y-error bars show SD values around the mean, *n* = 3. ^#^Not statistically significant (*p* > 0.05).

Figure S2 Effect of flavonoid-enriched tomato extract addition on m-IC_cl2_ cell-line viability. **(A)** Apoptosis and cell viability were assayed with antibodies to annexin V and the active form of Caspase 3, along with a propidium iodide exclusion assay. **(B)** Proliferation, with an BrdU incorporation assay and Ki-67 antibody staining. Y-error bars show SD values around the mean, *n* = 3. ^#^Not statistically significant (*p* > 0.05).

Figure S3 Proposed model for flavonoid inhibition of gut barrier recruitment of dendritic cell (DCs) during inflammation. ▲ represents microbe-associated molecular pattern (MAMP). **(A)** The normal situation of inflammation in response to MAMP detection on the apical side of epithelial cells. DCs are shown migrating toward a chemokine gradient. **(B)** The situation with dietary flavonoid intervention.
