# Supplementary figures and images for: Flavonoids from Engineered Tomatoes Inhibit Gut Barrier Pro-inflammatory Cytokines and Chemokines, via SAPK/JNK and p38 MAPK Pathways
Source: Front Nutr. 2017 Dec 18;4:61. doi: 10.3389/fnut.2017.00061 (PMC5741681; doi:10.3389/fnut.2017.00061)

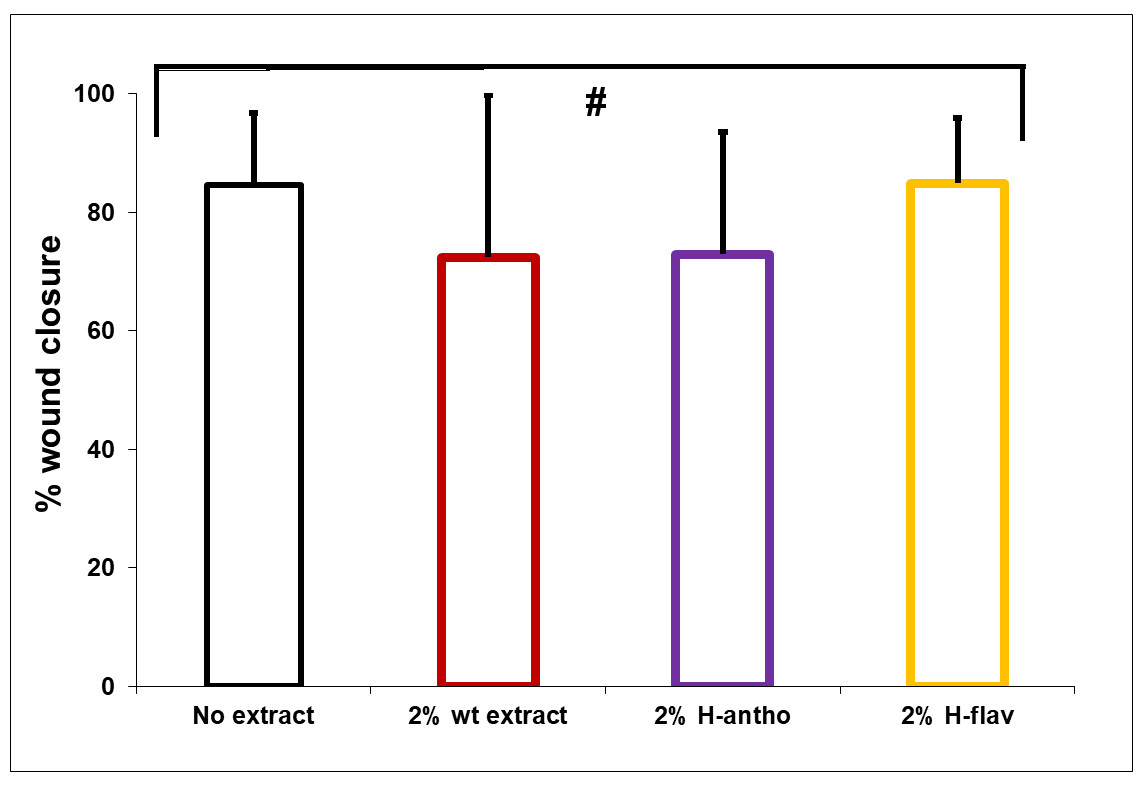

Supplement: Figure S1 — Effect of flavonoids on the extent of wound closure on an m-ICcl2 cell line-based scratch wound model, percentage closure calculated by wound diameter at T-0 compared with the same wound area at T-18 h using scaled wound width measurements. Y-error bars show SD values around the mean, n = 3. #Not statistically significant (p > 0.05). [file Image_1.jpeg]

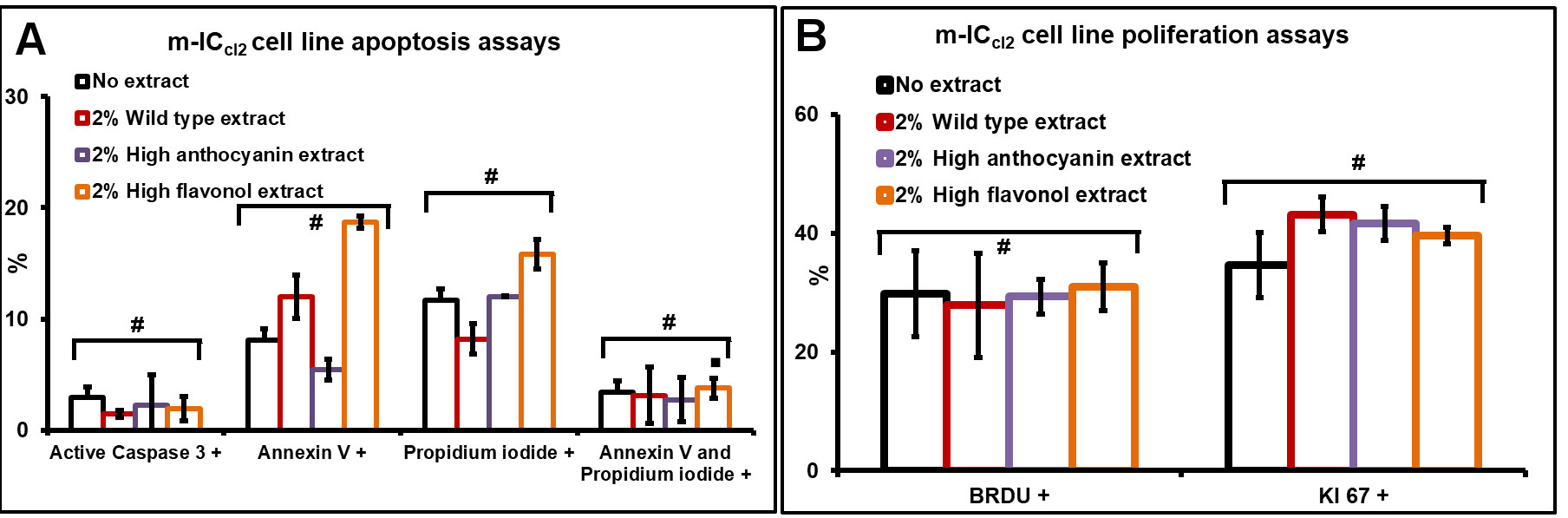

Supplement: Figure S2 — Effect of flavonoid-enriched tomato extract addition on m-ICcl2 cell-line viability. (A) Apoptosis and cell viability were assayed with antibodies to annexin V and the active form of Caspase 3, along with a propidium iodide exclusion assay. (B) Proliferation, with an BrdU incorporation assay and Ki-67 antibody staining. Y-error bars show SD values around the mean, n = 3. #Not statistically significant (p > 0.05). [file Image_2.jpeg]

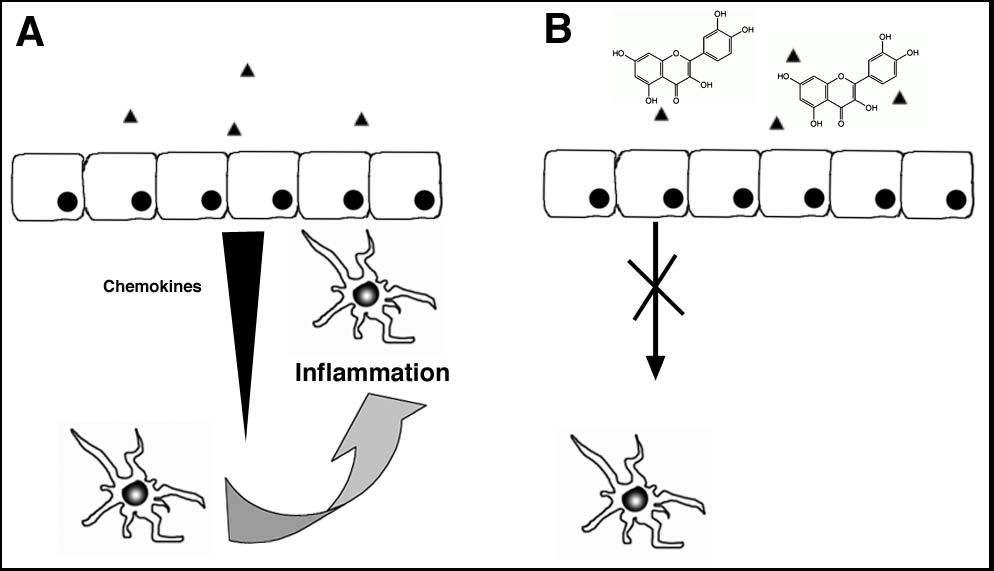

Supplement: Figure S3 — Proposed model for flavonoid inhibition of gut barrier recruitment of dendritic cell (DCs) during inflammation. ▲ represents microbe-associated molecular pattern (MAMP). (A) The normal situation of inflammation in response to MAMP detection on the apical side of epithelial cells. DCs are shown migrating toward a chemokine gradient. (B) The situation with dietary flavonoid intervention. [file Image_3.tiff]
